# Osteochondritis Dissecans of the Lateral Femoral Trochlea Healing after Isolated Medial Patellofemoral Ligament Reconstruction: A Case Report

**DOI:** 10.5704/MOJ.1907.006

**Published:** 2019-07

**Authors:** NC Barbosa, L Carvalho, LR Fernandes, D Castro, T Lino

**Affiliations:** Department of Orthopedics and Traumatology, Hospital Pedro Hispano, Matosinhos, Portugal

**Keywords:** osteochondritis dissecans, OCD, articular cartilage, patellofemoral instability

## Abstract

We report on a 12 years old female patient who had been diagnosed with patellofemoral instability – recurrent dislocation and anterior knee pain. Radiologic evaluation further revealed osteochondritis dissecans (OCD) of the lateral femoral trochlea. She underwent surgical procedure with isolated medial patellofemoral ligament reconstruction, no surgical procedure was done to the OCD lesion. Postoperatively, there was clinical improvement of patellofemoral instability, with radiological evidence of healing of the OCD lesion. Isolated realignment procedures such as medial patellofemoral ligament reconstruction may be associated with spontaneous healing of osteochondritis dissecans of the lateral femoral trochlea.

## Introduction

Osteochondritis dissecans (OCD) is an idiopathic acquired and focal lesion of the subchondral bone that can involve the articular cartilage. Repetitive microtrauma of the injured area may compromise healing. Patients with patellofemoral instability have a predisposition for chondral injury^1^. We present an osteochondritis dissecans of the lateral femoral trochlea, with spontaneous healing after isolated medial patellofemoral ligament reconstruction.

## Case Report

We report the case of a 12-year old female who had presented initially to a General Practitioner with right knee pain and patellofemoral instability.

**Fig. 1:**
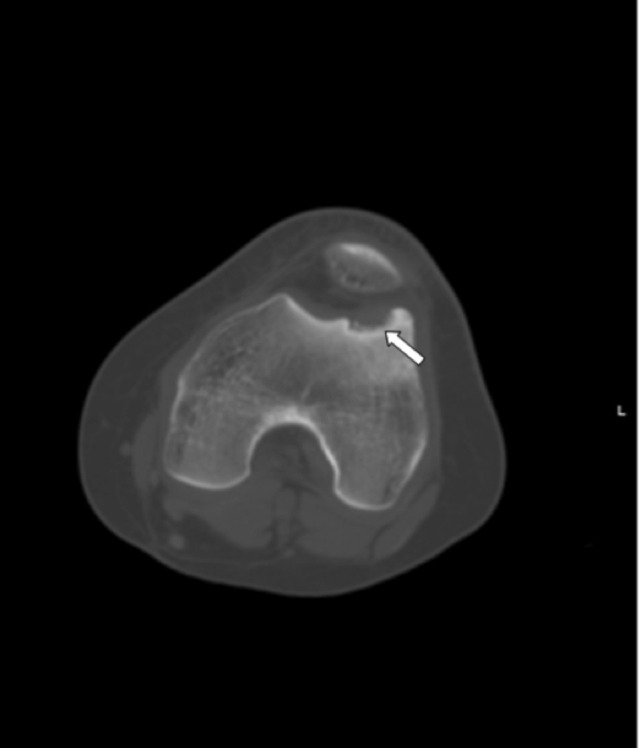
Preoperative computed tomography (CT) demonstrating lateral femoral trochlea OCD (white arrow).

She had undergone a CT scan of the knee in 2012, which had revealed an OCD lesion in the lateral femoral trochlea and patellofemoral joint instability (Fig. 1). Nonoperative management was attempted first with modification of the activity, intensive physical therapy program and knee brace with centralisation of the patella.

She was referred to us for orthopaedic consultation three years later in late November 2015. The patient complained mainly of anterolateral pain and instability of the right knee with more than three dislocation episodes per month. Physical examination showed diffuse tenderness anterolaterally, positive Rabot and Apprehension test. There was no lateral translation restriction.

**Fig. 2:**
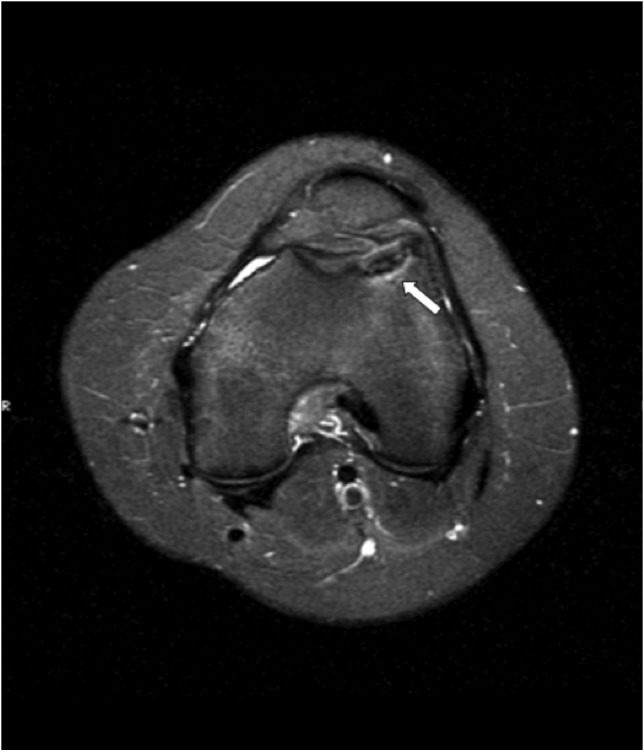
Preoperative magnetic resonance image demonstrates the fragment in situ with fluid signal and bone marrow edema under the lesion” (white arrow).

A preoperative MRI in 2015 (Fig. 2) demonstrated an unhealed OCD lesion, complete and undisplaced, surrounded with synovial fluid.

In early 2016, when she was 16 years of age, she underwent an isolated medial patellofemoral ligament (MPFL) reconstruction with Gracilis tendon, without lateral retinacular release^2^. No surgical procedure was performed on the OCD Lesion. There were no intra-operative or early postoperative complications.

At one year follow-up, she had no further episodes of dislocation, was satisfied with the surgery and clinically she presented with the following scores IKDC- 96.6; Kujala-98; Tegner-Lysholm-95. However, she experienced mild occasional anterior pain during strong activities.

**Fig. 3:**
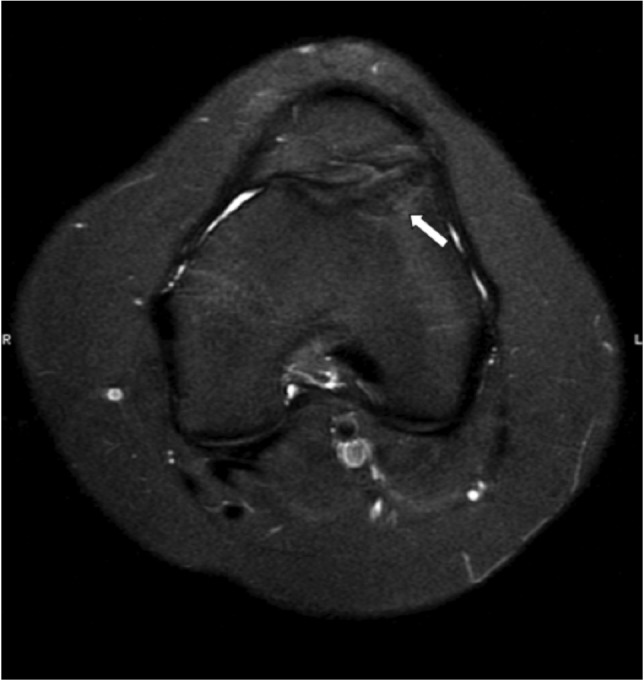
Postoperative magnetic resonance image demonstrates resolution of the bone marrow edema and continuity of the articular cartilage (white arrow).

A follow-up MRI in May 2017 (Fig. 3), revealed resolution of the lateral trochlea underlying bone marrow oedema, continuity of the overlying articular surface, and no clear demarcation line. Based on the report of Kramer *et al* we graded the OCD lesion as healed^1^.

## Discussion

To our knowledge this is the first report of an OCD lateral trochlea lesion healing after isolated MPFL reconstruction. OCD lesions usually are treated with cartilage restoration techniques such as isolated drilling, drilling and fixation, microfractures or autologous chondrocyte implantation.

Combination of cartilage restoration techniques and realignment techniques, to unload the treated lesion, are also common in femoral OCD.

Less commonly reported are realignment procedures for chondral injuries in patellofemoral OCD. Literature on the effects of realignment procedures for cartilage surgery is scarce, and instability is rarely mentioned in the treatment of lateral trochlea OCD as stated by Lording *et al*^3^.

Pidoriano *et al* have reported good-to-excellent subjective results in 23 patients with chondral defects at the inferior pole or lateral facet of the patella, after a Fulkerson tibial tubercle osteotomy^4^.

MPFL reconstruction is a surgical procedure indicated in patients with patellar instability. Dividing the MPFL has demonstrated increase in lateral facet peak contact pressure due to increased lateral patella tilt and translation^5^. Possibly, isolated MPFL reconstruction might have led to patellofemoral contact pressure changes which may have resulted in OCD healing.

In conclusion, isolated realignment of the extensor mechanism may favor healing of OCD lesions in the lateral femoral trochlea.
